# Artificial intelligence for symptom assessment and patient-reported outcomes in neuro-oncology

**DOI:** 10.1007/s11060-026-05832-9

**Published:** 2026-10-21

**Authors:** Lalanthica V. Yogendran, Chace Avery, Rita C. Crooms, Ahmad Ozair, Cara Siegel, Irene Guterman, John Y. Rhee, Jason Binder, Maria Minor, Laura Alder, Joshua Davis, Akanksha Sharma

**Affiliations:** 1 Department of Neurology & Rehabilitation Medicine, University of Cincinnati, Stetson Building 260 Stetson Street, Suite 2300, Cincinnati, OH 45267-0525 USA; 2 Department of Neurology, Icahn School of Medicine at Mount Sinai, New York City, NY USA; 3 Brookdale Department of Geriatrics and Palliative Medicine, Icahn School of Medicine at Mount Sinai, New York City, NY USA; 4 Department of Neurology and Neurosurgery, Montreal Neurological Institute-Hospital, McGill University, Montreal, QC Canada; 5 Departments of Medicine and Neurology, University of California, Los Angeles, CA USA; 6 University of Virginia Comprehensive Cancer Center, Charlottesville, VA USA; 7 Center for Neuro-Oncology, Department of Medical Oncology, Dana-Farber Cancer Institute, Boston, MA USA; 8 Division of Adult Palliative Care, Department of Supportive Oncology, Dana-Farber Cancer Institute, Boston, MA USA; 9 Department of Neurology, Harvard Medical School, Boston, MA USA; 10 Ember Health Inc., Greensboro, NC USA; 11 Department of Neurosurgery, Duke University Hospital, Durham, NC USA; 12 Division of Medical Oncology, Duke Cancer Institute, Durham, NC USA; 13 Department of Neurology, University of California Davis, Davis, CA USA; 14 Department of Neurology, Sutter Health/CPMC, San Francisco, CA USA

**Keywords:** Brain tumors, Glioma, Patient experience, Symptom monitoring, Machine learning, Neurological deficits

## Abstract

**Purpose:**

Patient-reported outcomes (PROs) are critical tools for measuring how patients experience their illness and treatment. Neuropsychiatric symptoms, headaches, seizures, and neurological deficits evolve over time and impact quality of life. In this narrative review, we summarize current literature on PROs in neuro-oncology, extant artificial intelligence (AI) tools for PRO analysis, considerations for real-world application, and ethical implications.

**Methods:**

We searched PubMed/MEDLINE, Embase, and Google Scholar for English-language articles published from January 2015 through April 2026, supplemented by reference-list reviews. Four themes were agreed upon, and authors were assigned to each theme. Authors conducted a literature review, selected articles, and synthesized the information under each of these themes. An initial 78 articles were narrowed down to 53 confirming eligibility if they reported PRO or symptom data in people with brain tumors or computational/AI methods were applied to PRO or symptom monitoring in oncology. Article selection was verified by two senior authors (L.V.Y, A.S.). Older, foundational studies were retained where they remain the primary evidence.

**Results:**

The growing volume and complexity of PRO data present challenges for traditional analysis methods. Integrating AI, including machine learning (ML) and large language models (LLMs), may help model longitudinal symptom trajectories to predict deterioration, extract symptoms from free-text clinical notes, and embed outputs in clinical decision support tools. Autonomy, informed consent, data security, bias, access, transparency and accountability should be considered.

**Conclusion:**

Successful translation will require interpretable, electronic health record-integrated, bias-mitigating tools that allow for actionable insights and preserve patient consent, privacy, and autonomy in this highly vulnerable population.

**Clinical trial number:**

Not applicable.

## Introduction

Artificial intelligence (AI) has emerged as a powerful tool in medicine, with great promise and equally significant concerns. Its application in neuro-oncology, including machine learning (ML) and large language models (LLM), remains nascent, particularly for symptom monitoring and patient reported outcomes (PRO) collection and analysis. This review addresses the application of AI to PRO and symptom data in people with brain tumors across the care continuum, including data collection and analysis, prediction of clinical trajectories and clinical decision support. AI applied to imaging, pathology, or genomics is outside its scope.

## Methods

### Search strategy

Each thematic section was assigned to 1–2 authors with relevant expertise who screened titles and abstracts and then full texts. We searched PubMed/MEDLINE, Embase, and Google Scholar for English-language articles published from January 2015 through April 2026, supplemented by reference lists from key articles and prior RANO-PRO publications. Search terms combined a neuro-oncology concept (e.g., “brain tumor,” “glioma,” “neuro-oncology,” “primary brain tumor,” “brain metastasis”) with a PRO/symptom concept (e.g., “patient-reported outcome,” “PRO,” “PROM,” “symptom burden,” “quality of life,” “ePRO”) and, where relevant, an analytic/AI concept (e.g., “artificial intelligence,” “machine learning,” “natural language processing,” “large language model,” “LLM,” “predictive model,” “clinical decision support”), joined using Boolean operators (AND/OR). Illustrative search strings included (“brain tumor” OR glioma OR “neuro-oncology”) AND (“patient-reported outcome*” OR PROM OR “symptom burden”) and (“large language model*” OR “machine learning” OR “artificial intelligence”) AND (“symptom” OR “patient-reported outcome*”) AND oncology.

Inclusion criteria were: (i) original research, systematic or scoping reviews, consensus guidance, or conference abstracts with extractable data; (ii) involving PRO, caregiver-reported or clinician-documented symptom data and (iii) either a brain tumor population or computational/AI methods applied to such data in oncology where neuro-oncology evidence was absent. Editorials without data, non-English articles, and studies without human symptom data were excluded. Selections were reviewed for relevance and consistency by two senior authors (L.V.Y., A.S). Findings were synthesized narratively. No formal risk of bias assessment was performed.

### Organization

The review is structured thematically with findings from these reviews organized under four focus areas: (1) PRO data quality and existing PRO instruments/frameworks in neuro oncology; (2) computational and AI/ML approaches to analyzing PRO and symptom data; (3) real world and emerging applications including considerations for patients and caregivers; (4) ethical considerations specific to using AI with PRO data in this population.

## Background on PROs in neuro oncology

Table 1 summarizes the most commonly used disease-specific health-related quality of life (HRQOL) and symptom PRO measures (PROMs) in neuro-oncology [1–4]. AI models are only as reliable as the PRO data on which they are trained. In neuro-oncology, missing data, selection bias, variable assessment timing, and clinician–patient discordance can distort symptom trajectories, treatment-effect estimates, and model predictions.

**Table 1:** PRO Instruments used in neuro oncology (1–5). *(Created Using Microsoft Word)*

Instrument	Focus	Select benefits	Select limitations
EORTC QLQ-C30 + QLQ-BN20 ((EORTC Quality of Life Questionnaire)	Cancer specific, measures function, symptoms, global quality of life BN20 is the brain tumor specific module	Most widely used PRO system in neuro oncology trials and provides comprehensive assessment	50-items can be burdensome for patients with neurological and cognitive disability, especially in later stages of disease
FACT-Br (Functional Assessment of Cancer Therapy-Brain)	General well being plus brain tumor specific cognitive, neurologic, functional, symptom related concerns	Greater emphasis on psychosocial quality of life	Less sensitive treatment related toxicities; 50-items resulting again in high patient burden and high attrition
MDASI-BT (MD Anderson Symptom Inventory)	Symptom severity and interference measured	Brief, 24 h recall period validated in primary brain tumor and brain metastases patients	Strictly measures interference and not broader psychosocial well being

The Response Assessment in Neuro-Oncology (RANO)-PRO Working Group has issued recommendations for selecting and applying PROs in neuro-oncology research, and the World Health Organization’s International Classification of Functioning, Disability, and Health has been used to evaluate instrument content validity [6, 7]. In 2020, RAN.

PRO proposed priority constructs for high-grade glioma trials and clinical care: pain, communication difficulty, perceived cognition, seizures, symptomatic adverse events, physical functioning, and role functioning [6, 8].

Clinician-rated measures including the Karnofsky Performance Status, Neurologic Function Score, and Common Terminology Criteria for Adverse Events remain integral to neuro-oncology, while the Mini Mental State Examination and Montreal Cognitive Assessment performance-based cognitive screening tests [9, 10]. Both complement PROs but may miss important symptoms and perceived neurocognitive changes. Clinicians underestimate symptom frequency and severity, while cognitive impairment, aphasia, and reduced insight can compromise self-report; caregiver proxies help but add interpretive complexity [11].

PRO completion declines with respondent burden, clinical deterioration, and logistical barriers; in a phase III trial of prophylactic cranial irradiation in extensive-stage small cell lung cancer (EORTC 22993/08993), fewer than half of expected quality-of-life forms were returned at 12 months [10]. Attrition is consequential because patients with worsening disease are more likely to stop responding, reducing statistical power and biasing results [12, 13]. Standardized schedules, electronic collection, reminders, staff education, compliance monitoring, and appropriate missing data methods may improve data quality.

## AI and machine learning contributions

### Importance of large-scale PRO datasets

Randomized trials in general oncology show that frequent longitudinal symptom monitoring can improve outcomes beyond those achieved through clinical encounters alone. In a single-institution RCT of 766 patients receiving chemotherapy for metastatic cancers (not neuro-oncology specific), electronic PRO monitoring with automated nurse alerts reduced emergency department visits, prolonged time on chemotherapy, and improved one-year survival [14]. The PRO-TECT cluster randomized trial (52 practices, *n* = 1,191) reproduced improvements in PROs at scale, with weekly electronic PRO surveys improving physical function, symptom control, and HRQOL at three months across diverse community oncology settings [14,15]. In neuro-oncology, evidence remains at the feasibility stage: a glioblastoma smartphone app pilot demonstrated uptake of longitudinal health outcome tracking, including caregiver-proxy entries, while a study of 43 patients with glioblastoma found that electronic PROMIS and EORTC assessments could be completed in a clinical setting [16, 17]. Cognitive impairment, neurologic decline, digital access, and variable engagement still warrant adaptations and evaluation of sustained completion; feasibility should not be mistaken for evidence of the clinical benefits observed in general oncology.

### Computational approaches to PRO analysis: from classical machine learning to large language models

Analyzing PRO datasets at scale requires computational methods that capture longitudinal patterns across heterogeneous symptom domains [18, 19]. ML, a branch of AI, uses algorithms that learn patterns from data rather than following explicit rules. Early neuro-oncology work applying ML to predict HRQOL after low grade brain tumor resection showed support vector machine and random forest classifiers could achieve areas under the curve above 0.90 for most symptom outcomes using routine perioperative inputs [20]. However, a systematic review of ML approaches to cancer symptom prediction found temporal and longitudinal modeling underrepresented, with neuro-oncology absent [21]. Natural language processing (NLP) enables extraction of structured information from clinical free text, the predominant and largely untapped component of the medical record. NLP has been used across oncology for symptom characterization, illness trajectory modeling, and goals-of-care documentation, although neuro-oncology specific applications remain limited [22]. These methods often require extensive manual rule development and domain-specific expertise, limiting adaptability across settings [23].

LLMs may support multiple stages of the care continuum [24]. Identifying illness phase transitions, often preceded by symptom changes that cross-sectional assessments fail to detect, is a central challenge in neuro-oncologic palliative care. Applied to 51,541 neuro-oncology clinical notes in the electronic health record (EHR), GPT-4o extracted nine symptom domains with mean sensitivity and specificity of 0.97, revealing 3.92 symptoms per note and prevalence rates substantially exceeding those in comparable clinical trials [25]. Patients receiving immune checkpoint inhibitors bore the highest symptom burden illustrating treatment-stratified insights that complement prospective PRO instruments. Whether these findings translate into robust, PRO-driven trajectory detection in primary brain tumor patients remains unknown.

Despite a roughly 20-fold increase in ML publications involving PRO data between 2011 and 2021, a 2024 systematic review of 52 oncology studies (searched through December 2022) found fewer than 8% externally validated their models and fewer than 6% shared reproducible code [26]. A 2025 scoping review of 94 studies using PROM data to predict patient outcomes across clinical fields found that only 10 were externally validated and three had reached an implementation stage; it also identified inconsistent handling of missing data and limited patient or clinician involvement in model development [27]. These findings suggest methodological and implementation gaps persist, although the two reviews differ in scope and do not represent a direct time trend. This validation deficit is consequential in neuro-oncology, where disease heterogeneity and modest sample sizes already constrain generalizability.

### Predictive analytics for symptom exacerbations, treatment response, and end-of-life phase transitions

Predictive models have evolved from regression to the AI-based methods outlined above, increasingly electronic PRO’s (ePROs), EHR-derived variables, imaging, and biologic data. Rigorous training, validation, and monitoring remain essential. These models may help anticipate clinical trajectories, offer more timely support, avoid excessive or futile treatment, and give patients and families time to prepare.

Models predicting treatment response, symptom exacerbation, and end-of-life transitions are less common in neuro-oncology than in oncology broadly. Symptoms are difficult to capture when neurologic deficits impair communication or use of digital tools, while symptoms such as seizures and neglect may be underrecognized and underreported [28]. Complementary data from caregivers, wearable devices, and EHR documentation are therefore vital [29]. Building on LLM-based symptom extraction from the EHR, multimodal ML could synthesize heterogeneous data streams into predictive insights [25].

### Integration into clinical decision support systems (CDSS)

Once a predictive model performs well, clinical integration is the harder step. In a recent review of cancer symptom-management CDSS, only 25.5% were EHR-integrated and 18.2% incorporated AI, with most rule-based rather than predictive [30].

Cooley et al.‘s integration of a cancer symptom management CDSS into two EHRs offers several lessons [31]. Symptom-management logic should be built around data reliably available in the EHR, ideally using standardized terminology. Alerts should be high-yield and paired with an explicit action, as high-volume, low-specificity alerts breed fatigue. Decisions resting on unreliable data or carrying harm if incorrect should require clinician verification. These lessons favor a controlled, auditable system over a fully automated one.

Tian et al. designed an autonomous agentic LLM workflow to detect cognitive concerns in unstructured clinical notes to support earlier memory evaluation, using five agents with defined roles: one classified notes, while others examined missed and incorrectly flagged cases and refined the classification prompt [32]. The model’s performance was comparable to, but did not surpass, an expert-refined workflow on an independent validation set, and the modular architecture made prompt refinement easier to inspect, offering a scalable model for interpretable clinical AI.

## Real-world applications and studies in neuro-oncology-based palliative settings

### Clinical integration

While AI and ML can identify symptom patterns and predict clinical trajectories, their value depends on translation into care delivery.

**Fig. 1:**
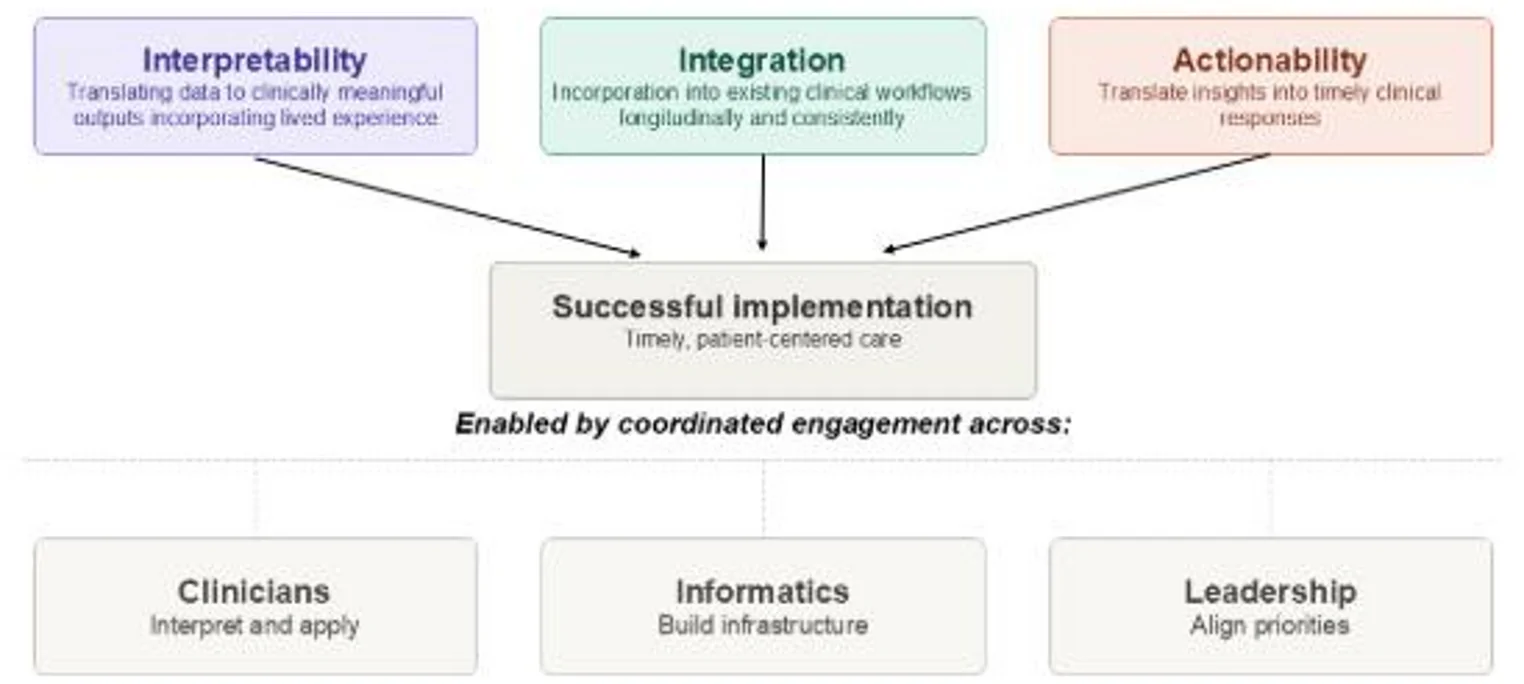
Framework for clinical integration of AI-generated PRO insights. *(created with Microsoft PowerPoint*,* converted to TIF format)*

A useful framework for implementation includes three interdependent components: interpretability, integration, and action [33]. Successful implementation requires coordinated engagement across clinical, informatics, operational, and health system leadership [34] (Fig. 1) to ensure AI-enabled workflows are scalable, interoperable, and sustainable rather than assembled ad hoc.

### Current state of integrated models

Deployed predictive modeling and personalized care CDSS are beginning to emerge in oncology. OncoPRO embeds the PRO version of the Common Terminology Criteria for Adverse Events into patients’ secure online portals, giving clinical teams real-time feedback to support earlier detection and management of immunotherapy toxicity [35]. Its alerts are triggered by predefined symptom thresholds, rather than an AI model; illustrating how established electronic PRO workflows can provide a foundation for later AI integration. Risk of 180-day mortality has also been used as a proxy for end-of-life phase transition. Xu et al. used patient-reported symptom data to predict 180-day mortality in advanced cancer, supporting symptom burden as an early marker of deterioration, while Manz et al. developed integrated an EHR-based 180-day mortality model to flag outpatients who may benefit from earlier advance care planning or palliative referral [36, 37].

Within neuro-oncology, most efforts are nascent. Cleveland Clinic has opened a glioblastoma remote monitoring study pairing Apple Watch biometric data with patient- or caregiver-entered symptoms to explore symptom predictors and clinical outcomes (NCT06129760, Registration Nov 2023). Investigators intend to use ML to analyze relationships between wearable signals and reported symptoms or clinical events. In France, the NEURO-PRO-GLIO study will evaluate the Resilience PRO platform in patients with IDH-mutant glioma to monitor neurologic symptoms, treatment tolerance, and quality of life (NCT07439302, Registration Feb 2026). Its registered protocol describes a predefined alert algorithm for severe or worsening symptoms, not a ML model; both studies may nonetheless supply data for future predictive models.

### AI-derived communication patterns and decision-making

A promising application of AI is to capture the patient and caregiver experience, outside hospital walls and scheduled clinic visits. Binder et al. reported an accessible, patient- and caregiver-facing digital support tool trained on peer-reviewed literature for glioblastoma [38]. Their retrospective analysis of deidentified conversation logs over a four-month period spanned 15,197 messages from multiple countries and languages; conversations frequently centered on imaging interpretation, fear of progression, treatment decision-making, symptom management, clinical trial support, caregiver distress, and end-of-life concerns.

This descriptive analysis characterized what users discussed; it did not test whether changes in their language predict clinical transitions. Prospective studies are needed to determine whether patient and caregiver language can reliably identify shifts in autonomy, decision-making burden, symptom escalation, caregiver takeover, or need for earlier palliative care.

## Ethical concerns regarding the use of AI tools for symptom assessment and management

Several ethical principles are especially salient for people with brain tumors, given the high incidence of cognitive deficits.

### Autonomy and informed consent

Decision-making capacity and autonomy are challenging in the care of patients with brain tumors, because cognition and neurological status may change over time [39]. Patient autonomy is further affected when LLMs shape or replace PROs (e.g., by inferring symptoms from a conversation with a clinician). A PRO is, by definition, reported directly by the patient without interpretation by a clinician or anyone else; an LLM-derived interpretation is therefore not a PRO and should be labeled and evaluated as a separate, AI derived measure. Surrogate-completed reports analyzed by AI may further distance the data from the patient’s own responses. Healthcare professionals should be able to see the original patient or proxy report, identify its source, and review clinically consequential AI interpretations against the underlying data and clinical context. Informed consent is important for preserving autonomy, and these patients may require a surrogate or proxy [39]. Healthcare organizations and multidisciplinary implementation teams should evaluate platforms, document intended uses and limitations, and provide clinicians with concise training and escalation pathways. Individual clinicians need not become AI experts. Clinicians can then explain to patients when AI is used, what information it draws on, how its output may affect care, and what alternatives are available. Patients expect clear communication about the purpose of collected data and their rights to data transparency and security [40].

### Data security and privacy

Healthcare-related AI tools access substantial volumes of data, including PROs, posing privacy risks such as an AI model being misled to release protected health information (PHI). In the United States (US), the Health Insurance Portability and Accountability Act (HIPAA) establishes privacy and security requirements for PHI held by covered entities and their business associates. Section  3060 of the 21st Century Cures Act excludes certain clinician-facing clinical decision support software functions from the definition of a medical device overseen by the US Food and Drug Administration (FDA), provided specified criteria are met, including that clinicians can independently review the basis for recommendations [41]. This is not a blanket exemption for AI or EHR-embedded tools, nor does it exempt software from privacy, institutional, or other oversight. Rapidly developed software may contain unvalidated algorithms, provide misleading recommendations, or lack adequate data safeguards.

Outside the US, requirements differ. In the European Union (EU), the General Data Protection Regulation (GDPR) governs the processing of personal data, including health data, and places conditions on certain automated decisions with significant effects. The EU AI Act establishes a risk-based framework under which qualifying AI enabled medical devices may be classified. The applicable requirements depend on the tool’s intended use and regulatory status. Privacy protections and device oversight should therefore be assessed in the jurisdiction where a tool is developed or deployed, rather than inferred from US rules.

### Bias and access

How users develop, apply, and interpret AI outputs may perpetuate or worsen existing disparities in diagnosis, care access, treatment, and outcomes by race, ethnicity, gender, and socioeconomic status [42, 43]. Data bias arises from non-representative study design and data selection: clinical trial populations underrepresent female and non-White patients, and two-thirds of US trial-supporting institutions are urban or socioeconomically advantaged [44, 45]. Clinician-generated text used to train LLMs also reflects implicit bias, with stigmatizing language varying by race, gender identity, and behavioral factors [46]. Black, Asian, Hispanic, and non-native English-speaking patients are less likely to receive symptomatic and palliative treatment; symptom burden documentation is also affected by bias [47–49]. Algorithmic bias can emerge through aggregation and feature selection and proxy variables can obscure true relationships between groups [50]. Models have underestimated healthcare resource needs for Black patients based on historically lower spending rather than actual need [50]. Model deployment bias occurs when users defer uncritically to model outputs rather than applying independent clinical judgment, allowing errors to persist and compound [51]. This risk is greater in AI-generated summaries, as representing a whole population even when they reflect only a narrow or skewed subset.

### Balancing risk and benefit

Despite these risks, AI models can be highly effective with careful, iterative evaluation. Oh et al. proposed adding to traditional bioethical principles a fifth principle of explicability, requiring that AI systems be understandable and their limitations clearly communicated [52]. Rhee et al., drawing on qualitative interviews with patients, care-partners, clinicians, and bioethicists in neuro-oncology, identified key themes: maintaining primacy of the physician-patient relationship, recognizing uniquely human capacities AI lacks, ensuring clinician oversight, and prioritizing patient education [40]. Specific to bias, Mehari et al. proposed a consensus “HEALS” framework of five equity-centered principles for AI across the neuro-oncology continuum [53]. Across these frameworks, a unifying principle emerges: AI implementation must remain patient-centered and committed to equity, privacy, and humane care, supported by formal oversight that includes risk-benefit assessment and mechanisms for redress.

## Conclusion

### Strengths and limitations

This review brings together evidence on PROs, AI methods, clinical implementation, and ethics in neuro-oncology, while distinguishing neuro-oncology specific findings from oncology more broadly. However, the literature spans heterogeneous populations, measures, technologies, and study designs, and narrative selection may not represent all relevant work. Because no formal quality or risk-of-bias assessment was performed, the review cannot establish the overall quality of the publications or compare strength of evidence across approaches. Findings from small feasibility studies, retrospective analyses, protocols, and studies outside neuro-oncology should therefore be treated as signals of potential rather than evidence of clinical benefit in patients with brain tumors.

### Key takeaways

Patient-reported outcomes and AI could make neuro-oncology more patient-centered and data-driven, but current evidence is limited by small studies, missing and inconsistent symptom data, and weak external validation. Progress will depend on multicenter collaboration, standardized PRO frameworks, interoperable data systems, and broader inputs such as caregiver reports, wearables, and clinical narratives.

Successful translation will require tools that are interpretable, EHR-integrated, and able to deliver actionable insights in real time, while preserving clinician oversight. Ethical implementation must prioritize informed consent, privacy, autonomy, and bias mitigation so these systems support, not replace, clinical judgment in a highly vulnerable population.

## Data Availability

No datasets were generated or analysed during the current study.
